# Insomnia and sleep-disordered breathing in FKRP-related limb-girdle muscular dystrophy R9. The Norwegian LGMDR9 cohort study (2020)

**DOI:** 10.1007/s00415-023-11978-7

**Published:** 2023-09-11

**Authors:** Synnøve Jensen, Karin Abeler, Oddgeir Friborg, Assami Rosner, Caroline Olsborg, Svein Ivar Mellgren, Kai Ivar Müller, Andreas Dybesland Rosenberger, Monica L. Vold, Kjell Arne Arntzen

**Affiliations:** 1 National Neuromuscular Centre Norway and Department of Neurology, University Hospital of North Norway, 9038 Tromsø, Norway; 2 Department of Clinical Medicine, Faculty of Health Sciences, University of Tromsø-The Artic University of Norway, Tromsø, Norway; 3 Department of Neurology and Neurophysiology, University Hospital of North Norway, Tromsø, Norway; 4 Department of Psychology, Faculty of Health Sciences, University of Tromsø-The Artic University of Norway, Tromsø, Norway; 5 Department of Cardiology, University Hospital of North Norway, Tromsø, Norway; 6 Department of Neurology, Sørlandet Hospital Trust, Kristiansand, Norway; 7 Department of Respiratory Medicine, University Hospital of North Norway, Tromsø, Norway

**Keywords:** Muscular dystrophies, limb-girdle, Sleep initiation and maintenance disorders, Respiration disorders, sleep apnea syndromes, Sleep, Fatigue

## Abstract

Limb-girdle muscular dystrophy R9 (LGMDR9) is a progressive and disabling genetic muscle disease. Sleep is relevant in the patient care as it impacts on health, functioning, and well-being. LGMDR9 may potentially affect sleep by physical or emotional symptoms, myalgia, or sleep-disordered breathing (SDB) through cardiorespiratory involvement. The objective was to investigate the occurrence of insomnia and unrecognized or untreated SDB in LGMDR9, associated factors, and relationships with fatigue and health-related quality of life (HRQoL). All 90 adults in a Norwegian LGMDR9 cohort received questionnaires on sleep, fatigue, and HRQoL. Forty-nine of them underwent clinical assessments and 26 without mask-based therapy for respiration disorders additionally underwent polysomnography (PSG) and capnometry. Among 77 questionnaire respondents, 31% received mask-based therapy. The prevalence of insomnia was 32% of both those with and without such therapy but was significantly increased in fatigued respondents (54% vs 21%). Insomnia levels correlated inversely with mental HRQoL. Among 26 PSG candidates, an apnea–hypopnea index (AHI) ≥ 5/h was observed in 16/26 subjects (≥ 15/h in 8/26) with median 6.8 obstructive apneas and 0.2 central apneas per hour of sleep. The AHI was related to advancing age and an ejection fraction < 50%. Sleep-related hypoventilation was detected in one subject. Fatigue severity did not correlate with motor function or nocturnal metrics of respiration or sleep but with Maximal Inspiratory Pressure (r = − 0.46). The results indicate that insomnia and SDB are underrecognized comorbidities in LGMDR9 and associated with HRQoL impairment and heart failure, respectively. We propose an increased attention to insomnia and SDB in the interdisciplinary care of LGMDR9. Insomnia and pulmonary function should be examined in fatigued patients.

## Introduction

Limb-girdle muscular dystrophy type R9 (LGMDR9) is a rare autosomal recessive muscle disease caused by pathogenic variants in the fukutin-related protein (FKRP) gene. LGMDR9 is characterized by slowly progressive proximal weakness and may be accompanied by cardiomyopathy and/or ventilatory failure [1–3]. Currently, there is no causal treatment available. Clinical management is interdisciplinary, focusing on supporting cardiorespiratory function, preventing complications, and optimizing daily functioning and health-related quality of life (HRQoL). Sleep affects overall health and HRQoL [4]. A previous study on the Norwegian LGMDR9 cohort (Jensen SM et al., submitted paper) showed that HRQoL was impaired and subjective sleep disturbance more frequent compared to studies on general populations. Additionally, fatigue was prevalent and closely related to disease burden. According to an explanatory model in neuromuscular disorders (NMD), sleep disturbance, together with physical inactivity and pain, may act as a perpetuating factor of fatigue [5]. Sleep may thus be an area that needs increased clinical attention in LGMDR9. Sleep disturbance is unspecific but may represent sleep disorders with specific treatment options such as insomnia and sleep-related breathing disorders (sleep-disordered breathing, SDB), which both are relevant in NMD.

Insomnia disorders involve nighttime problems of initiation or maintenance of sleep or early-morning awakening that causes daytime impairment or dissatisfaction with sleep [6]. Insomnia relates to various somatic and mental health conditions [7]. First-line treatment is Cognitive Behavioral Therapy for Insomnia (CBT-I) and both physical and digital CBT-I have been found effective [7]. Studies on patients with comorbid insomnia and sleep apnea (COMISA) have shown that CBT-I with an adapted and interdisciplinary approach may also improve tolerance and adherence to mask-based therapy [8]. In Duchenne muscular dystrophy, the increased risk of insomnia and its potentially negative influence on essential non-invasive ventilation (NIV) and HRQoL have been recognized and CBT-I or sleep hygiene advices are recommended interventions [9]. A recent study on milder muscular dystrophies including a subgroup with LGMD reported that insomnia and fatigue are related [10]. We are not aware of any other studies of insomnia in patients with LGMD. The impact of insomnia in NMD in general seems to be understudied.

SDB includes sleep-related hypoventilation and hypoxemia, obstructive sleep apnea (OSA), and central sleep apnea (CSA) [6]. NMD may cause hypoventilation by respiratory muscle weakness, scoliosis, stiffening of the chest wall, and subsequent intrapulmonary changes [11]. Furthermore, NMD may promote OSA by pharyngeal hypotonia, macroglossia, or possibly by collapsibility of the upper airways due to low lung volumes and may promote CSA by cardiomyopathy or hypoventilation [12]. Hypoventilation during rapid eye movement (REM) sleep is recognized as the earliest sign of respiratory failure [12], which may be related to sleeping position, impairment of chemosensitivity during sleep, and physiological REM sleep atonia [13, 14]. Mask-based therapies may provide efficient support for SDB, whereas inappropriate treatment can have aggravating effects [14]. In Duchenne muscular dystrophy and amyotrophic lateral sclerosis, which are more rapidly progressive diseases, non-invasive ventilation (NIV) has also been shown to prolong survival and improve HRQoL [14, 15]. In LGMD, respiratory involvement is rather unexplored and no disease-specific respiratory guidelines exist.

Our previous study of natural history in the Norwegian LGMDR9 cohort [3] showed that indication for mask-based therapy was restrictive pulmonary function alone in 47%, OSA alone in 22%, and both combined in 31%. Initiation of mask-based therapy was usually preceded by wheelchair dependency except in those who only had OSA. Females were more prone to become wheelchair dependent and require mask-based therapy, whereas males were more predisposed to cardiomyopathy. The level of respiratory follow-up was variable, which means that SDB may be underrecognized.

In the present study, we assessed the prevalence and levels of insomnia and unrecognized or untreated SDB in the Norwegian LGMDR9 cohort and examined relationships with demographic and clinical variables and indicators of HRQoL, particularly fatigue. We also examined whether fatigue in people with LGMDR9 relates to pulmonary function since respiratory muscle weakness and chest wall changes tend to increase the work of breathing. More knowledge about these issues may optimize the clinical management of this patient group.

## Methods

### Participants

**Fig. 1:**
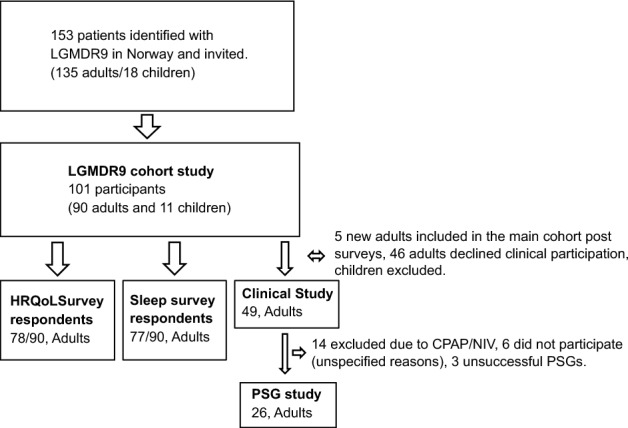
Flow chart. *PSG* polysomnography, *CPAP* continuous positive airway pressure, *NIV* non-invasive ventilation

Previously, we identified 153 individuals (135 adults, ≥ 16 years) with a genetically confirmed LGMDR9 in Norway [3]. They were all invited to participation in «The Norwegian LGMDR9 cohort study» at the National Neuromuscular Centre, Norway (NMK), University Hospital of North Norway (UNN). Participants provided clinical information by completing a project-specific questionnaire and by consenting to retrieve patient notes from the specialist centers. All adult participants in the cohort study were invited to respectively a sleep survey, a HRQoL survey, and a clinical observational study consisting of a 2-day visit at UNN for examinations according to a study protocol. Clinical participants without mask-based therapies for respiration disorders were additionally invited to a polysomnography (PSG) recording. The present study includes data from the two survey studies, the clinical study, and the PSG study (Fig. 1).

### Procedure

The surveys were administered by regular mail. The sleep survey (consisting of instruments 1–4 described below) was administered 4 weeks after the HRQoL survey (consisting of instruments 5 and 6 below). The Fatigue Severity Scale and the respiratory questionnaire were also completed during the hospital visit. The schedule for the clinical study included capillary blood gas, clinical neurological examination, Body Mass Index (BMI), echocardiography with semi-automated estimation of the biplane left ventricular ejection fraction (EF), the 32-item Motor Function Measure, and pulmonary function tests (Table 1). For eligible participants, PSG with capnometry was performed within the frame of the clinical study. PSG equipment was attached in the afternoon by a technician at the Department of Clinical Neurophysiology, and the recording was conducted ambulatory at the patient hotel. SDB included sleep-related hypoventilation, sleep-related hypoxemia, and sleep apnea (SA) as defined below. EF was reported as EF < 50% (impaired) or ≥ 50% (normal). Data from the clinical study were collected and managed using REDCap^1^electronic data capture tools hosted at UNN [16, 17].

**Table 1:** The schedule for the clinical participants

Day 0	Check in at the patient hotel
Day 1	Capillary blood gas (7.30 a.m.)
	Weight/height
	Self-report instruments
	Electrocardiography
	Muscle ultrasound
	Motor tests
	Mounting of PSG (15 p.m.)
Day 2	Detachment of PSG (7.30 a.m.)
	Pulmonary lab
	Echocardiography
	Clinical neurological examination
	Cognitive test
	Motor tests

*PSG* polysomnography

### Self-report instruments

1. Fatigue: The Norwegian version of Fatigue Severity Scale (FSS) comprises nine items which are rated on a seven-point Likert scale 1–7 [18]. A mean item score ≥ 5 indicates clinically significant fatigue, based on recommendations in a Norwegian validation study [19]. Missing values were not replaced.
2. Excessive daytime sleepiness (EDS): The Norwegian version of Epworth Sleepiness Scale (ESS) includes eight items with a 0–3 response range representing low to high chance of dozing off in a given situation [20, 21]. A sum score > 10 indicates significant EDS. Missing values were not replaced.
3. Insomnia: The Bergen Insomnia Scale (BIS) was originally developed complying to the criteria for chronic insomnia of the fourth edition of the Diagnostic and Statistical Manual of Mental Disorders (DSM-4) [22], and subsequently adjusted to the criteria of DSM-5/the third edition of the International Classification of Sleep Disorders (ICSD-3) [23]. The BIS contains six items (score range 0–7) indicating how frequently, i.e., days a week, the patient has experienced nighttime (three items) and daytime (two items) insomnia symptoms, and non-restorative sleep (one item). The time frame is the last three months. The minimum criterion for insomnia is a score of ≥ 3 on both one nighttime and one daytime item. The sum score of all six items (range 0-no to 42-maximum) indexes the level of symptom burden as a continuous score. Missing values were not replaced.
4. Sleep-related problems: Quality-of-life questions for patients on home mechanical ventilation (HMV) (here: «respiratory questionnaire») originate from a Swedish stress research program [24] and subsequent research on HRQoL in patients with chronic alveolar hypoventilation [25, 26] and implementation in the Swedish [27] and the Norwegian national register for patients on HMV [28]. It contains five items, and a previous study with NIV intervention showed that all items except daytime tiredness correlated with morning PaCO2 levels, which also related to generic HRQoL [26]. The translated Norwegian version used in the present study was not validated, and the severity scale in terms of frequency differed slightly from the Swedish version: never or almost never/sometimes/several times a week (Swedish version: once a week)/daily or almost daily. Missing values were not replaced.
5. HRQoL: The Norwegian version of the 36-item Short Form Health Survey (SF-36) version 1 [29] is a generic measure. The items are scored on a 2 to 6-point categorical scale aggregated into nine 0–100-point (100 = maximal HRQoL) subscales: physical functioning, role limitations due to physical problems, bodily pain, general health, vitality, social functioning, role limitations due to emotions, mental health, and change in health over the past year. Missing values were replaced according to the scoring algorithm.
6. HRQoL: The Norwegian version of the Individualized Neuromuscular Quality of Life questionnaire (INQoL) version 2.0 [30] is a disease-specific measure comprising seven symptom domains (i.e., muscle weakness, myalgia, fatigue, myotonia, diplopia, ptosis, and dysphagia), five life domains (i.e., activities related to daily living/leisure/work, independence, social relationships, emotions, and body image), and two treatment domains. The items are scored on a 7-point scale (Likert type, but each number also has a categorical description) transformed into separate domain scores, and an aggregated score (the INQoL index) as a proxy of disease burden or impact on HRQoL. The INQoL index is based only on the items on impact and impact importance of each life domain and does not include the items of the life domains that describe the perceived status. Each transformed score range 0–100 (100 = maximal burden). Missing values were replaced according to the scoring algorithm.

### Pulmonary function tests (PFT)

Spirometry was performed according to The American Thoracic Society and the European Respiratory Society technical guidelines 2019 [31]. In addition to the standard sitting position, spirometry was also performed in the supine position. This has been recommended for patients with NMD since diaphragmatic dysfunction tends to cause a relatively low performance in the supine position [32]. We calculated the relative drop in Forced Vital Capacity (FVC) from sitting to supine position (∆FVC), and FVC percent predicted in both positions (FVC% and FVC% supine), using reference equations for standard position from The Global Lung Function Initiative (GLI) 2021 [33]. Maximal inspiratory pressure (MIP) was performed in a sitting position and measured three times, but additional one or two times when the technique was inadequate. The best of repeated measurements was used and recorded as percent predicted (MIP%) using reference equations from GLI [34].

### The 32-item motor function measure (MFM32)

MFM32 is a clinician-reported quantitative scale of motor function in individuals with NMD [35]. The scale is adapted to all degrees of severity; for walking and non-walking patients. Each item is scored on a 4-point Likert scale from 0 (cannot initiate the task) to 3 (performs the task fully). Total score ranges from 0 to 96 points, based on three subdomain scores: D1—Standing and transfers (39), D2—Axial and proximal motor function (36), and D3—Distal motor function (21). The result is expressed as percentage of the maximum possible score. The assessors were MFM32-certified physiotherapists with special expertise in neuromuscular disease.

### PSG study

SOMNOscreen equipment and Domino version 2.7.0 software (Somnomedics, Randersacker, Germany) were used for PSG. PSG recording was performed in accordance with the guidelines of The American Academy of Sleep Medicine (AASM) version 2.4 (2017) [36]. Six electroencephalographic leads (F3/F4, C3/C4, O1/O2), right and left electrooculography, and submental electromyography were used for sleep scoring. Pressure flow oral-nasal cannula, inductive thoracic and abdominal belts, and oximetry (Nonin) were used for respiratory assessment. Body position was monitored by an accelerometer incorporated in the PSG device attached to the chest. PSG scoring was divided between two consultant clinical neurophysiologists at UNN (co-authors) and conducted according to AASM [36]. Total sleep time (TST), sleep efficiency, amount of REM sleep and deep (N3) sleep (minutes and %TST), time spent in supine position (%TST), and mean oxygen saturation (SpO_2_) were reported and included in the analyses. Desaturation was defined as a ≥ 3% decrease in SpO_2_. Apnea was scored when there was ≥ 10 s duration of ≥ 90% air flow reduction, and classified as obstructive, central, and mixed [36]. Hypopnea was scored when there was ≥ 10 s duration of ≥ 30% air flow reduction associated with a ≥ 3% decrease in SpO2 and/or an EEG arousal [36] and were not classified considering both the general risk of misclassification of hypopneas [37] and the additional risk in NMD where events related to respiratory muscle weakness may resemble or compound obstructive or central events [12, 14]. Oxygen desaturation index (ODI), apnea–hypopnea index (AHI), apnea index (AI), obstructive AI (oAI), central AI (cAI), and microarousal index (MAI) were calculated as events per hour of sleep. AHI was additionally calculated differentially in Non-REM (NREM) and REM sleep, and in supine and non-supine position, and the respective ratios were reported among candidates with SA. We defined SA as AHI ≥ 5 independent of symptoms and comorbidities. SA severity was defined as mild (AHI 5.0–14.9), moderate (AHI 15.0–29.9), or severe (AHI ≥ 30.0) [36]. Sleep-related hypoxemia was defined as SpO2 ≤ 88% for > 5 consecutive minutes in accordance with ICSD-3 (AASM 2014) [6], but we used the definition regardless of concurrent capnometry outcomes. Additionally, we assessed time spent with SpO2 < 90% (minutes and %TST) and the presence of Cheyne-Stokes respiration.

SenTec Digital Monitoring System (SenTec AG, Therwil, Switzerland) was used for nocturnal transcutaneous PCO2 (PtcCO2) monitoring. The sensor was placed on the forehead. Current AASM criteria for sleep-related hypoventilation were applied: an increase in PtcCO2 to a value > 7.33 kPa (55 mmHg) for ≥ 10 min and/or ≥ 1.33 kPa (10 mmHg) increase in PtcCO2 during sleep in comparison to awake supine values to a value exceeding 6.67 kPa (50 mmHg) for ≥ 10 min [38]. Mean and maximal PtcCO2, and maximal rise in PtcCO2 from initial values were used as variables.

### Statistical analyses

Data were analyzed using IBM SPSS Statistics for Windows (Version 28.0. Armonk, NY: IBM Corp.). Distribution of continuous data are described using median and interquartile range (IQR). Categorical variables are presented with frequencies. Simple group comparisons from continuous variables were performed with independent t-test with bootstrapping (5000 resamples). Group comparison from categorical variables were assessed with Pearson chi-square or Fischer exact test with mid p-correction, as appropriate, and significant findings presented with odds ratio (OR) with confidence interval (CI). Correlations were examined with Spearman rank correlation (r_s_) or Pearson correlation (r) according to the distributions and inspected with scatter plot and curve estimation. In the assessment of predictors of AHI, multiple linear regression (with backward elimination method) was used due to several relevant relationships with potential confounding effects: age, sex, and BMI as well-established risk factors for SA [39] and among LGMDR9-related variables (pulmonary function indices, EF < 50%, macroglossia, and dysarthria or dysphagia), EF < 50% was found relevant based on p value in t test (p < 0.10). Assumption of normal distribution of the residuals was assessed with P–P plot, skewness, and kurtosis, homoscedasticity by scatter plotting predicted versus residual values, and influential cases in terms of Cook’s distance and DeltaFit. The alpha level was set to p < 0.05. Considering the exploratory nature of the study, correction for multiple testing was not used in this study [40].

## Results

### Participants

Figure 1 provides an overview of the recruitment and inclusion of the participants. In total, 77/90 (86%) adults participated in the sleep survey, and the response rate of the four instruments ranged 72–75/90 (80–83%). The response rate of the HRQoL survey was 78/90 (87%). The inclusion rate for the clinical study was 49/95 (52%). Comparable levels of age, sex distribution, wheelchair dependency, and established mask-based therapies indicated that the samples participating in the two surveys and the clinical study were representative of the cohort. In the clinical study, 35/49 (14/22 females and 21/27 males) did not receive mask-based therapy and were thus eligible for PSG. However, six participants (five females and one male) either declined the invitation to PSG or were not invited due to practical inconvenience, and three recordings (of one female and two males) were unsuccessful, hence excluded. The 26 successful PSG registrations represented 8 of 14 (57%) eligible females and 18 of 21 (86%) eligible males.

### Sleep and HRQoL surveys

Background data and outcomes of the sleep survey are presented in Table 2. There was a comparable number of female and male participants. The subgroup with mask-based therapies comprised eight respondents with continuous positive airway pressure support (CPAP) (i.e., 10% of all respondents) and 16 with NIV (i.e., 21% of all respondents) as bi-level positive airway pressure or ventilator, of which five also used NIV at daytime. The same subgroup had a female preponderance (63% vs 38%), was older (median age 56 vs 37 years), and had a higher prevalence of wheelchair dependency (67% vs 17%).

**Table 2:** Background data and outcomes of the sleep survey in participants without vs with mask-based therapies

	All n = 72–77	No mask n = 50–53	Mask n = 21–24
	n (%) or M (IQR)	n (%) or M (IQR)	n (%) or M (IQR)
Females	37 (48)	22 (42)	15 (62.5)
Males	40 (52)	31 (58)	9 (37.5)
Age (years)	47 (32–58)	37 (27–54)	56 (50–63)
Daily smokers	4 (5)	3/53 (6)	1 (4)
COPD	1 (1)	1/53 (2)	0 (0)
W/C	25 (33)	9/52 (17)	16 (67)
ESS (0–24)	4.0 (2.0–8.0)	5.0 (2.0–8.0)	3.5 (0.0–8.0)
EDS (ESS > 10)	7 (10)	6 (12)	1 (5)
BIS (0–42)	9 (4–16)	9 (5–17)	8 (4–15)
Insomnia	23 (32)	16 (31)	7 (32)
SOL^a^	20 (27)	16 (31)	4 (18)
WASO^a^	14 (19)	6 (12)	8 (36)
Early awakening^a^	16 (22)	11 (22)	5 (23)
Non-restorative sleep^a^	35 (49)	27 (53)	8 (38)
Dissatisfaction w/sleep^a^	16 (22)	11 (22)	5 (23)
Daytime tiredness^a^	27 (37)	19 (37)	8 (36)
Respiratory questionnaire			
Several awakenings^b^	31 (43)	19 (38)	12 (55)
Morning headache^b^	7 (10)	5 (10)	2 (9)
Refreshing sleep^b^	46 (64)	32 (64)	14 (64)
Daytime tiredness^b^	29 (40)	21 (42)	8 (36)
Nocturnal dyspnea^b^	2 (3)	2 (4)	0 (0)
FSS (1–7)	4.3 (3.6–5.7)	4.2 (3.3–5.5)	4.7 (3.9–6.2)
Fatigue (FSS ≥ 5)	29 (40)	18 (36)	11 (48)

*M* median, *IQR* inter-quartile range, *COPD* chronic obstructive pulmonary disease, *W/C* wheelchair dependency, *ESS* Epworth Sleepiness Scale, *EDS* excessive daytime sleepiness, *BIS* Bergen Insomnia Scale, *SOL* sleep onset latency (> 30 min), *WASO* wake after sleep onset (> 30 min), *FSS* Fatigue Severity Scale

^a^≥ 3 days a week for the last 3 months

^b^Several times a week or more

**Fig. 2:**
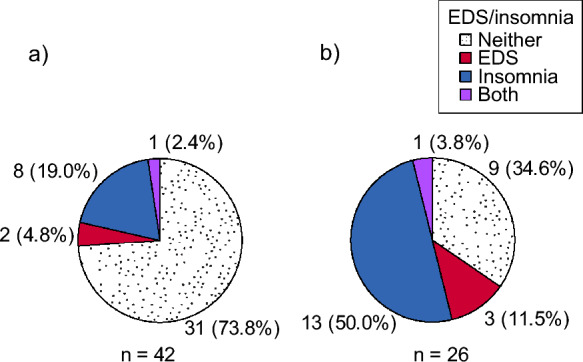
Prevalence of insomnia and excessive daytime sleepiness (EDS) among LGMDR9 participants **a** without fatigue (Fatigue Severity Scale (FSS) < 5) and **b** with fatigue (FSS ≥ 5)

In total, 32% had insomnia and 10% EDS. Overlap of insomnia and EDS occurred in two respondents (Fig. 2). The prevalence of fatigue or frequent daytime tiredness ranged 37–40%. Frequent nightly awakenings and/or non-restorative sleep were common (36–49%), whereas 10% reported frequent morning headache and 3% frequent nocturnal dyspnea.

Insomnia was equally prevalent in the subgroup with nocturnal masks as in those without. Only one patient with mask-based therapy had EDS, i.e., severe residual sleepiness. Compared to the subgroup without masks, those who used masks showed a tendency towards a higher prevalence of nightly awakenings (55% vs 38%) and wake after sleep onset (> 30 min) (36% vs 12%) but a relatively lower prevalence of sleep onset latency (18% vs 31%). Prevalence of early awakening and dissatisfaction with sleep in the two groups was comparable (22%).

The prevalence of insomnia was 36% in females and 27% in males (p = 0.40) and unrelated to wheelchair dependency (p = 0.81). Level of insomnia symptoms was not correlated with age (r_s_ = 0.07) or physical or social aspects of HRQoL but to poorer outcomes on mental aspects of HRQoL, especially vitality, pain, and fatigue (Table 3). Correspondingly, the prevalence of insomnia was increased in fatigued compared to non-fatigued patients; 54% vs 21%; (OR 4.41, CI 1.52–12.79, p = 0.005) (Fig. 2). Concurrently, the prevalence of EDS was relatively low (10%) and not significantly increased in fatigued patients (p = 0.30) (Fig. 2).

**Table 3:** Correlations between levels of insomnia (the sum score of Bergen Insomnia Scale) and health-related quality of life (HRQoL) in LGMDR9

HRQOL Subscales	r_s_ (n = 68–70)	95% CI	p
SF-36 physical	0.19	− 0.06, 0.41	0.13
SF-36 role physical	− 0.20	− 0.42, − 0.05	0.11
SF-36 bodily pain	− 0.41	− 0.60, − 0.19	0.0004
SF-36 general health	− 0.22	− 0.44, 0.03	0.07
SF-36 vitality	− 0.46	− 0.64, − 0.25	< 0.0001
SF-36 social	− 0.18	− 0.40, 0.07	0.14
SF-36 role emotional	− 0.35	− 0.55, − 0.11	0.003
SF-36 mental	− 0.38	− 0.57, − 0.15	0.0012
INQoL muscle weakness	− 0.03	− 0.27, 0.22	0.48
INQoL fatigue	0.40	0.17, 0.58	0.0007
INQoL myalgia	0.30	0.06, 0.50	0.01
INQoL activities	0.02	− 0.22, 0.26	0.86
INQoL independence	− 0.03	− 0.27, 0.21	0.80
INQoL relationships	0.006	− 0.24, 0.25	0.96
INQoL emotions	0.26	0.02–0.47	0.03
INQoL index	0.09	− 0.16, 0.32	0.48

Negative relationships with SF-36 and positive with INQoL mean that increasing levels of insomnia are associated with poorer HRQoL outcomes. Only subscales considered relevant in relation to sleep were included

*r*_*s*_ Spearman’s rho, *CI* confidence interval, *SF-36* 36-item Short Form Health Survey, *INQoL* Individualized Neuromuscular Quality of Life questionnaire

### PSG study and clinical study

Background data and outcomes of the PSG study and additional assessments of the 26 PSG candidates during their hospital visit are shown in Table 4. Females and males had comparable age and BMI, but females showed relatively poorer outcomes on pulmonary function tests and a higher proportion of males had an impaired EF. Two subjects were wheelchair dependent. The PSG candidates with indications of SDB, except mild SA, are presented individually in Table 5. One of the PSG candidates, a male with comorbid chronic obstructive pulmonary disease and an EF < 50% had tried mask-based therapy previously but not tolerated it (Table 5, Subject 1). PSG showed severe SA, and he was the only PSG candidate with Cheyne-Stokes respiration and a SpO2 < 90% exceeding 5% TST. He also met the criteria for insomnia in the survey, which may have contributed to the lack of tolerance to the mask. Nineteen PSG candidates had a successful capnometry. Mean PtcCO2 levels were median 5.69 kPa in females and 5.99 kPa in males and the highest mean value recorded was 6.62 kPa. Maximum PtcCO2 levels were median 6.13 kPa in females and 6.58 kPa in males and the highest level recorded was 7.25 kPa (Table 4). Only one met the applied criteria for sleep-related hypoventilation (Table 5, Subject 9). No correlation between PtcCO2 levels and PFT for any of the variables included in the study (see method section) was found. None met the applied criterion for sleep-related hypoxemia. SA was detected in 16/26 PSG candidates (4/8 females and 12/18 males) and was moderate or severe in 8/26 (Table 4). Among the 16 subjects with SA, median oAI was 6.8/h and cAI 0.2/h and the oAI comprised > 50% of AHI in one half, whereas the hypopneas predominated in the other half. A cAI ≥ 5 was only recorded in one subject. This subject had an implanted cardiac resynchronization therapy defibrillator and an EF < 50% but also a predominant, severe OSA (Table 5, Subject 2).

**Table 4:** Background data and outcomes of the polysomnography (PSG) study and additional assessments of the PSG candidates during their hospital visit

	Females (n = 8) n (%) or median	Males (n = 18) n (%) or median	All (n = 26) Range
Characteristics			
Age (years)	40	41	16–64
BMI (kg/m^2^)	26.2	26.5	21.0–36.4
BMI > 30 kg/m^2^	1 (0)	3 (17)	–
Daily smokers	1 (13)	1 (6) N.D.: 1	–
W/C	1 (13)	1 (6)	–
Self-report instruments			
FSS (1–7)	4.4	4.1	2–6
Fatigue (FSS ≥ 5)	3 (38)	6 (33)	–
Respiratory questionnaire			
Several awakenings^a^	2 (25)	7 (41)	–
Morning headache^a^	1 (13)	1 (6)	–
Refreshing sleep^a^	4 (50)	10 (56)	–
Daytime tiredness^a^	6 (75)	8 (44)	–
Nocturnal dyspnea^a^	0 (0)	0 (0)	–
Cardiac/pulmonary function			
FVC%	72	89	60–111
FVC% sup	63 N.D.:1	85 N.D.:4	32–103
∆FVC (%)	22 N.D.:1	11 N.D.:4	4–33
MIP%	62	81 N.D.:1	28–108
EF < 50%	1 (13)	7 (41) N.D.: 1	–
PSG study			
TST (hours:min)	6:31	6:44	4:50–8:57
Sleep efficiency (%)	92.0	90.2	63.0–97.6
Supine (%TST)	65.1	45.6	3.3–100.0
REM sleep (hours:min)	1:05	1:10	0:01–1:10
REM sleep (%TST)	15.6	17.0	0.0–28.5
N3 sleep (hours:min)	1:30	1:18	0:0–2:31
N3 sleep (%TST)	22.6	21.2	0.0–36.9
ODI (n/hour)	2.3	7.6	0.0–50.1
AHI (n/hour)	6.6	10.2	0.2–63.6
AI (n/hour)	1.8	3.6	0–42.4
oAI (n/hour)	1.3	1.5	0–34.7
cAI (n/hour)	0.0	0.3	0–7.3
AHI 5.0–14.9/hour	2 (25)	6 (33)	–
AHI 15.0–29.9/hour	2 (25)	4 (22)	–
AHI ≥ 30.0/hour	0 (0)	2 (11)	–
MAI (n/hour)	22.3	54.1	6.8–91.9
Mean SpO2 (%)	97	96	93–98
Mean PtcCO2 (kPa)	5.69 N.D.: 3	5.99 N.D.: 4	5.23–6.62
Max PtcCO2 (kPa)	6.13 N.D.: 3	6.58 N.D.: 4	5.48–7.25

*BMI* body mass index, *N.D.* no data, *W/C* wheelchair dependency, *FSS* Fatigue Severity Scale, *FVC%* forced vital capacity percent predicted, *FVC% *_*sup*_ FVC in supine position percent of predicted FVC in standard position, *∆FVC* relative FVC drop from sitting to supine position, *MIP%* maximal inspired pressure percent predicted, *EF* left ventricular ejection fraction, *TST* total sleep time, *REM* rapid eye movement, *N3* non-REM sleep stage 3, *ODI* oxygen desaturation index, *AHI* apnea–hypopnea index, *MAI* microarousal index, *SpO2* oxygen saturation, *PtcCO2* transcutaneous carbon dioxide tension

^a^Several times a week or more

**Table 5:** Characteristics and measurements of the polysomnography candidates with hypoventilation or moderate/severe sleep apnea

Subject no	1	2	3	4	5	6	7	8	9
Age (years)	62	55	46	51	59	57	44	49	31
Sex	M	M	M	F	M	F	M	M	M
BMI (kg/m^2^)	27.2	25.6	22.9	23.4	26.8	24.5	28.5	23.8	21.9
W/C	+	−	−	−	−	+	−	−	−
COPD	+	−	−	−	−	−	−	−	−
EF < 50%	+	+^a^	−	+	−	−	+	−	− (50%)
Respiratory metrics									
SA	+	+	+	+	+	+	+	+	−
AHI n/hour	43.3	63.6	21.6	21.4	20.7	20.6	16.3	16.9	1.4
AI n/hour	3.9	42.4	9.4	3.8	18.0	11.0	11.4	13.7	1.1
oAI n/hour	0.3	34.7	9.4	3.8	18.0	10.9	8.8	12.4	1.1
cAI n/hour	0.3	7.3	0.0	0.0	0.0	0.2	1.7	1.2	0.0
ODI n/hour	41.2	50.1	18.2	3.7	10.2	4.0	7.8	7.7	0.5
CSR	+	−	−	−	−	−	−	−	−
SpO2 < 90% min (%TST)	72 (16)	15 (4)	6 (1)	3 (< 1)	3 (1)	1 (< 1)	4 (1)	1 (1)	3 (< 1)
HV	−	−	−	−	N.D	−	−	−	+
FVC%	42	85	107	88	88	75	100	115	104
FVC%_sup_	32	N.D	99	63	N.D	73	87	103	88
∆FVC (%)	25	N.D	8	28	N.D	4	13	10	16
MIP%	54	N.D	107	59	94	81	92	81	65
Symptoms									
Several awakenings^b^	+	+	−	−	−	−	+	−	−
Morning headache^b^	+	−	−	−	−	−	−	−	−
Refreshing sleep^b^	−	−	+	+	+	+	+	+	+
Daytime tiredness^b^	−	+	−	+	+	+	−	−	+
Nocturnal dyspnea^b^	−	−	−	−	−	−	−	−	−
FSS (1–7)	4.1	5.9	1.9	4.4	4.1	3.7	1.8	2.3	4.2

*M* male, *F* female, *BMI* body mass index, *W/C* wheelchair dependency, *COPD* chronic obstructive pulmonary disease, *EF* ejection fraction, *SA* sleep apnea (AHI ≥ 5), *AHI* apnea–hypopnea index, *AI* apnea index, *oAI* obstructive AI, *cAI* central AI, *ODI* oxygen-desaturation index, *CSR* Cheyne-Stokes respiration, *SpO2* oxygen saturation, *TST* total sleep time, *HV* sleep-related hypoventilation, *N.D.* no data, *FVC%* forced vital capacity percent predicted, *FVC%*_*sup*_ FVC in supine position percent of predicted FVC in standard position, *∆FVC* relative FVC drop from sitting to supine position, *MIP%* maximal inspired pressure percent predicted, *FSS* Fatigue Severity Scale

^a^Implanted cardiac resynchronization therapy defibrillator

^b^Several times a week or more

All four PSG candidates with a BMI > 30 kg/m^2^ and all eight with EF < 50% had SA. AHI was relatively increased in those with an EF < 50% compared to those with a normal EF (p = 0.096). AHI was uncorrelated with the PFT metrics (r_s_ = 0.18 to − 0.06, p ≥ 0.40) and unrelated to macroglossia (n = 4) and dysarthria and/or dysphagia (n = 3). Multiple regression showed that AHI correlated with advancing age and an EF < 50% but not with BMI or sex (Table 6). We assessed the relationships between AHI and sleeping position and REM sleep, respectively. Respiratory events tend to increase in the supine sleeping position and patients with LGMDR9 may be more prone to the supine position due to mobility difficulties. REM sleep tend to aggravate OSA and events related to respiratory muscle weakness due to physiological REM atonia, as mentioned, whereas central apneas usually occur in NREM sleep [41]. Among the subjects with SA, median AHI ratio supine/non-supine was 3.2 (range 1.3–16.4) and median AHI ratio REM/NREM was 2.7 (range 0–6.2).

**Table 6:** Multivariate regression model with stepwise backward elimination of non-significant predictors. The apnea–hypopnea index (AHI) from the polysomnography candidates as dependent variable

Dependent variable: AHI (n = 24)						
Model	R^2^/R^2^_adj_	Predictor	Beta	β	p^a^	95% CI for beta
1	0.544/0.453	EF < 50% (vs ≥ 50%)	13.25	0.43	0.019 (0.011)	2.46, 24.03
		BMI (kg/m^2^)	− 0.56	− 0.14	0.399 (0.820)	− 1.90, − 0.79
		Age (years)	0.59	0.56	0.002 (< 0.001)	0.24, 0.94
		Male (vs female)	− 1.36	− 0.04	0.789 (0.931)	− 11.77, 9.06
2	0.543/0.477	EF < 50% (vs ≥ 50%)	12.79	0.42	0.014 (0.007)	2.84, 22.74
		BMI (kg/m^2^)	− 0.51	− 0.13	0.411 (0.828)	− 1.78, 0.76
		Age (years)	0.58	0.55	0.002 (< 0.001)	0.25, 0.92
3	0.527/0.485	EF < 50% (vs ≥ 50%)	11.75	0.39	0.018 (0.005)	2.23, 21.26
		Age (years)	0.56	0.54	0.002 (< 0.001)	0.24, 0.90

Normality assumptions for the residuals were not met. Square root transformation of the AHI variable normalized the residuals and yielded similar findings as the untransformed model

*R*^*2*^ explained variance, *adj* adjusted, *beta* unstandardized regression coefficient, *β *standardized beta, *CI* confidence interval, *EF* ejection fraction, *BMI* body mass index

^a^p-value based on transformed data

**Fig. 3:**
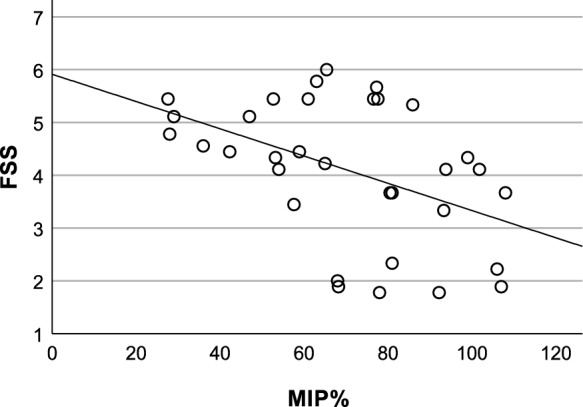
Scatter plot showing the relationship between Maximal Inspiratory Pressure percent predicted (MIP%) and Fatigue Severity Scale (FSS) (range 1–7, i.e., no to maximal fatigue) in the clinical participants without mask-based therapies

The AHI was unrelated to the items of the respiratory questionnaire. However, the two individuals with severe SA stood out with problems on three of five items (Table 5, Subject 1 and 2). No correlations between FSS and nocturnal measurements were detected including TST, amount N3 sleep (minutes and %TST), amount REM sleep (minutes and %TST), AHI, MAI, ODI, mean SpO2, and mean PtcCO2. Nevertheless, relationships between FSS and pulmonary function impairment were observed and most clearly between FSS and MIP% (Table 7, Fig. 3). In the subgroup with SDB, a correlation also between FSS and ∆FVC was found. In the whole group without mask-based therapies, FSS correlated both with MIP% and FVC % supine but not with MFM32 (D3: r = − 0.25, D2: r = − 0.20, D1: r = − 0.10, total score: r = − 0.15) and capillary PCO2, bicarbonate, or base excess. Comparatively, in the group with established mask-based therapies, no correlation between FSS and PFT metrics was detected and there was even a tendency of an inverse relationship between fatigue severity and ∆FVC (Table 7). This group consisted of four subjects with CPAP and ten subjects with NIV (one also partly daytime and another 24 hours) and had relatively higher levels of age, BMI, and wheelchair dependency as well as poorer outcomes on PFT compared to the group without mask-based therapy (Table 7).

**Table 7:** Characteristics, pulmonary function tests (PFT), and correlations between PFT and Fatigue Severity Scale (FSS) in clinical participants without vs with mask-based therapies

	No mask			Mask
	All no mask n = 35	PSG study n = 26	SDB^a^ n = 17	n = 14
Age (years), median	38	41	47	56
BMI (kg/m^2^), median	25.8	26.5	26.8	30.4
W/C, n (%)	6 (17)	2 (8)	2 (12)	10 (71)
FSS, median	4.3	4.2	4.1	4.6
FVC%, median	88	89	93	59 N.D.: 1
FVC% - FSS, r	− 0.27, p = 0.12	− 0.06, p = 0.78	− 0.07, p = 0.79	− 0.03, p = 0.93
FVC% sup, median	74 N.D.: 9	77 N.D.: 5	85 N.D.: 5	40 N.D.: 3
FVC% sup - FSS, r	− 0.39, p = 0.046	− 0.25, p = 0.27	− 0.35, p = 0.27	0.11, p = 0.74
∆FVC (%), median	12 N.D.: 9	11 N.D.: 5	11 N.D.: 5	19 N.D.: 3
∆FVC (%) - FSS, r	0.20, p = 0.34	0.37, p = 0.10	0.59, p = 0.043	− 0.53, p = 0.09
MIP% median	68 N.D.: 2	78 N.D.: 1	80 N.D.: 1	54 N.D.: 1
MIP% - FSS, r	− 0.46, p = 0.008	− 0.43, p = 0.030	− 0.54, p = 0.031	− 0.02, p = 0.94

r = Pearson correlation coefficient

*PSG* polysomnography, *SDB* sleep-disordered breathing, *BMI* body mass index, *W/C* wheelchair dependency, *FVC%* forced vital capacity percent predicted, *FVC%* supine FVC supine percent of predicted FVC in standard position, *N.D.* no data, *∆FVC* relative FVC drop from sitting to supine position, *MIP%* maximal inspired pressure percent predicted

^a^PSG candidates where SDB was detected:16 with sleep apnea and one with hypoventilation according to the applied criteria

## Discussion

In this study of a national cohort with LGMDR9, insomnia was prevalent in both subjects with and without mask-based therapies and significantly more prevalent among fatigued compared to non-fatigued patients. Insomnia severity correlated negatively with mental HRQoL. The PSG study uncovered a high occurrence of previously unrecognized SA, which was predominated by obstructive apneas in 50% and by unclassified hypopneas in the remaining and was related to the supine sleeping position and to REM sleep. Central apneas were relatively infrequent and only one had Cheyne-Stokes respiration. AHI was correlated with increasing age and an impaired EF. One subject met the AASM criteria for nocturnal hypoventilation. Fatigue severity was unrelated to nocturnal measurements of sleep and respiration, morning capillary blood gas, and motor function but negatively correlated with pulmonary function, particularly inspiratory muscle strength.

### Insomnia

Insomnia was more prevalent relative to two previous Norwegian population studies (32% vs 20%) [23, 42]. Co-existing EDS and insomnia was rare, which resonates with previous knowledge that sleepiness is usually not present in insomnia [7]. Potential contributors to an increased prevalence of insomnia in LGMDR9 are immobility, myalgia, mental distress, and SA [8, 43–45]. This study showed that levels of insomnia were associated with mental aspects of HRQoL including pain. Previously, we found that both mental, physical, and social aspects of SF-36, but not pain, were significantly poorer in our LGMDR9 cohort compared to a reference population (Jensen SM et al., submitted paper). Insomnia was also related to the mental aspects SF-36 vitality and INQoL fatigue and the increased prevalence of insomnia in fatigued compared to non-fatigued patients substantiates this relationship. This finding aligns with the aforementioned study in slowly progressive muscular dystrophies [10] and with a study of a large clinical sample with insomnia [46]. In the latter study, a bidirectional relationship was proposed and depression was identified as a significant mediator. Worth noting is that CBT-I is also recommended for comorbid insomnia, particularly as it may potentially improve accompanying conditions such as pain or depression [7].

Patients who received mask-based therapy also had a high prevalence of insomnia, but the nocturnal pattern differed such that there were less difficulties with sleep initiation and more excessive wake time throughout the night, compared to patients without a mask. However, early morning awakening was comparable in both subgroups. Insomnia in these patients may reflect inappropriate mask-based therapy or the need for additional intervention for insomnia. As aforementioned, insomnia in patients with mask-based therapy is a particular concern since it may affect adherence and, resultantly, treatment outcomes. The potential need for interdisciplinary treatment in comorbid SDB and insomnia may be a limiting factor.

### Sleep-disordered breathing (SDB)

The diagnosis of SDB relies on objective measurements of respiratory functions during sleep. Symptoms of untreated or sub-optimally treated SDB may reflect in the respiratory questionnaires, gauging of morning headache and nocturnal dyspnea, and the ESS assessing EDS. Although EDS has heterogeneous causes, it is commonly associated with SDB [47]. The survey showed that the prevalence of these potential symptoms of SDB was low. EDS was even relatively low compared to a previous Norwegian general population study (9.6% vs 17.7%) [48].

AHI was elevated in a high proportion of our PSG candidates. This was not unexpected considering the high prevalence in general populations [39]. Furthermore, applying the specified criteria for sleep-related hypoventilation and hypoxemia, only one mild case of sleep-related hypoventilation was detected. Nevertheless, as previous studies have demonstrated, this highly depends on the diagnostic criteria [49–51]. In Duchenne muscular dystrophy, disease-specific treatment criteria for hypoventilation, more liberal than AASM criteria, have been established, yet comparative trials remain [9, 51]. However, a low rate of sleep-related hypoventilation in our study can be explained by the sample. Compared to those with established mask-based therapy, the PSG sample represented a relatively younger age group and earlier stages of disease by a low rate of wheelchair dependency and more preserved pulmonary function. Additionally, the group with mask-based therapy had a relatively higher rate of obesity (median BMI > 30 kg/m^2^), which is an independent risk factor for hypoventilation (obesity-hypoventilation syndrome).

While the AHI in one half of those with SA was predominated by obstructive apneas, the other half had predominantly hypopneas, which could be obstructive, central, diaphragmatic, or compounded. AHI was related to advancing age, which is a general risk factor for OSA [39] but could also be related to progression of the disease. Concerning comorbid associations with SA, a Finnish nationwide registry-based case–control study found that SA (including OSA and CSA) is strongly associated with obesity, heart failure, and respiratory disease [52]. In concurrence, we found an association with heart failure. Previous studies have shown that heart failure is associated with both CSA and OSA and suggested that heart failure may promote OSA through CSA or upper airway edema [53], but the mechanisms have not been established. In the current study, central apneas were rare. Nonetheless, considering the unspecified nature of the hypopneas, this study cannot distinguish whether the correlation between AHI and an EF < 50% relates to a specific type of events. Further, the low BMI levels and relatively preserved pulmonary function among our PSG candidates, in general, may explain why BMI and pulmonary metrics did not turn out as significant correlates of the AHI. This may also indicate that SA is not underrecognized among patients with obesity or significant respiratory involvement since treated patients were not included in the PSG study. A report from a task force of the European Respiratory Society and the European Sleep Research Society advised to take the individual susceptibility into account in treatment decisions [54]. Comorbidities like heart failure, obesity, and respiratory muscle weakness may represent such vulnerabilities. Furthermore, SA treatment has shown potential to improve comorbidities such as obesity, metabolic disease, and heart failure [53, 55], which emphasizes the importance of considering morbidity profile in the monitoring of SDB.

AHI was related to the supine sleeping position and to REM sleep, which is well known in OSA [56] but could also be related to diaphragm weakness [14]. Since the sleeping position and the proportion of REM sleep during PSG registration may differ from habitual sleep without recording devices, these variables also need to be considered to avoid under or overdiagnosis. Additionally, the association with position means that positional therapy, preventing supine sleeping position, may be an option. Nevertheless, patients with muscle disease may need special consideration due to mobility or pain issues.

AHI was poorly correlated with sleep-related symptoms. However, research shows that SA is commonly sub-clinical, and both symptomatic and sub-clinical SA may be associated with cardiovascular disease [57]. This means that detection and intervention could be relevant independent of symptoms and consequently that regular sleep studies may be required in risk patients. Additionally, since symptoms and AHI are poor predictors of severity, there also exists a need for better biomarkers for treatment decisions [54].

Our study did not support the idea that fatigue in our LGMR9 cohort could be related to untreated SDB. However, we did find fatigue severity to be negatively correlated with inspiratory muscle strength (MIP). Since FSS was uncorrelated with metrics of blood gases and motor function, the mechanism of the FSS-MIP relationship seems more likely to be the work of breathing rather than an association with general motor function impairment or respiratory disturbances caused by the inspiratory weakness. The FSS-MIP relationship was absent in the group with established mask-based therapy. Potential explanations are the alleviation of breathing work due to ventilatory support and less physical exposure due to a higher rate of wheelchair dependency. Interestingly, a correlation between FSS and MIP has also been found in polio-myelitis [58] and several other studies have shown that MIP is a clinically meaningful outcome measure for NMD [59–61].

### Strengths and limitations

This study provides insights into the prevalence of insomnia and unrecognized SDB in LGMDR9 and clinically relevant relationships with these sleep disorders and with fatigue. Important strengths of the surveys were sample sizes and response rates. The clinical study included a representative sample of the cohort and integrated assessments with standardized methods. PSG and PCO2 monitoring are considered gold standard methods for diagnosing SDB.

The study also has several limitations. Due to the exploratory design, we accepted a higher risk of conducting type I error (false positive), and instead of correcting for multiple testing, we urge for caution in assigning significance to p-values in the range of 0.01 to 0.05. Findings that are flagged as significant require replication. The prevalence of chronic insomnia disorder may be underestimated as the BIS does not cover all daytime impairments included in ICSD-3 (e.g., cognitive impairment, mood disturbance, and impaired motivation) or overestimated since a clinical interview is required to rule out exclusion criteria [7], such as insomnia due to poor sleep environment or insomnia mimics like circadian rhythm problems or restless legs syndrome. The PSG sample size was low, which increases the risk of type II errors (false negative conclusions), and females were relatively underrepresented. Identification of individuals with SDB relied on diagnostic criteria, which are mostly based on expert opinions [49, 60]. PSG may overestimate AHI by its tendency to increase the time in supine position [61] or underestimate it by reducing the proportion of REM sleep [62]. Sleep during PSG may have been impacted by an unfamiliar sleeping environment and setting and myalgia or tiredness after physical tests. However, TST, percentage of N3 sleep, and sleep efficiency indicated successful registrations. Technical issues limited the data completeness on capnometry and supine spirometry. Lastly, the study was conducted during the COVID-19 pandemic. Although the data were collected in relatively normal periods, this may have impacted the subjective measurements (HRQoL, sleep, and fatigue).

## Conclusions

The study indicates that insomnia is prevalent in LGMDR9 and related to mental HRQoL. Correlations indicate the need for particular attention to insomnia in patients with fatigue, pain, or negative emotions since insomnia treatment may also relieve associated symptoms. The prevalence study also suggests a need for increased recognition of insomnia in patients receiving mask-based therapies as insomnia may affect device adherence and, consequently, treatment outcomes. While mask-based therapy was established in 31% of participants, SA was underrecognized among remaining participants, and identified risk factors were advancing age and an EF < 50%. Since SA treatment may benefit cardiac outcomes, sleep studies in patients with heart failure should be considered in particular. Fatigue was related to MIP but not motor function. This relationship advocates for pulmonary function tests in fatigued patients and suggests that MIP is a clinically meaningful measure in LGMDR9. More studies on respiratory natural history of LGMDR9 and biomarkers of SDB are required to decide proper indication and timing of pulmonary function tests, sleep studies, and treatment of SDB.

## Data Availability

The supporting data are not publicly available due to ethical restrictions. The participants have not given written consent for their data to be shared publicly.

## Footnotes

1 REDCap (Research Electronic Data Capture) is a secure, web-based software platform designed to support data capture for research studies, providing (1) an intuitive interface for validated data capture; (2) audit trails for tracking data manipulation and export procedures; (3) automated export procedures for seamless data downloads to common statistical packages; and (4) procedures for data integration and interoperability with external sources.
